# E-cigarette prevalence among Malaysian adults and types and flavors of e-cigarette products used by cigarette smokers who vape: Findings from the 2020 ITC Malaysia Survey

**DOI:** 10.18332/tid/146363

**Published:** 2022-03-31

**Authors:** Pete Driezen, Amer Siddiq Amer Nordin, Farizah Mohd Hairi, Anne Yee, Nur Amani Ahmad Tajuddin, Siti Idayu Hasan, Mahmoud Danaee, Ina Sharyn Kamaludin, Susan C. Kaai, Mi Yan, Matthew Grey, Anne C. K. Quah, Mary E. Thompson, Geoffrey T. Fong

**Affiliations:** 1 Department of Psychology, University of Waterloo, Waterloo, Canada; 2 School of Public Health Sciences, University of Waterloo, Waterloo, Canada; 3 Department of Psychological Medicine, Faculty of Medicine, University of Malaya, Kuala Lumpur, Malaysia; 4 Nicotine Addiction Research Group, University of Malaya Centre of Addiction Sciences, University of Malaya, Kuala Lumpur, Malaysia; 5 Department of Social and Preventive Medicine, Faculty of Medicine, University of Malaya, Kuala Lumpur, Malaysia; 6 Department of Primary Care Medicine, Faculty of Medicine, University of Malaya, Kuala Lumpur, Malaysia; 7 Department of Statistics and Actuarial Science, University of Waterloo, Waterloo, Canada; 8 Ontario Institute for Cancer Research, Toronto, Canada

**Keywords:** e-cigarettes, prevalence, Malaysia, flavors, nicotine

## Abstract

**INTRODUCTION:**

E-cigarettes (ECs) have become increasingly common in many countries, including Malaysia. The prevalence of EC use increased in Malaysia from 0.8% in 2011 to 4.9% in 2019. Three quarters of Malaysian EC users also smoke combustible cigarettes, and the prevalence of EC use among Malaysian smokers in 2014 was consistent with the prevalence of use among smokers from Canada and the US in 2016. The purpose of this study was to estimate the prevalence of EC use among Malaysian adults aged ≥18 years in 2020 and the types of EC products and flavors used by cigarette smokers who also used ECs at least monthly.

**METHODS:**

Data came from 1253 adults aged ≥18 years who participated in the 2020 International Tobacco Control Malaysia Wave 1 Survey. Weighted descriptive statistics were used to estimate the prevalence of adults who reported ever using ECs and the prevalence who used ECs either monthly, weekly, or daily. The types of EC products and flavors used were compared by frequency of EC use among current smokers who used ECs at least monthly (n=459).

**RESULTS:**

Overall, 5.4% (95% CI: 3.7–7.5) of Malaysian adults reported using ECs on a daily basis in 2020. Among current cigarette smokers who used ECs daily, 81.0% (95% CI: 72.5–87.7) used nicotine in their ECs, 46.2% (95% CI: 37.8–54.7) used pre-filled ECs, and 60.4% (95% CI: 51.9–68.6) reported being somewhat/very addicted to ECs. The most common EC flavors were fruit, coffee, and menthol/ mint.

**CONCLUSIONS:**

Continued surveillance of EC use is necessary to monitor EC use in non-tobacco using populations while longitudinal research is needed to determine the extent to which ECs are, or are not, related to quitting smoking.

## INTRODUCTION

E-cigarettes (ECs) have become more common in many countries^1-4^, especially among youth and young adults^3,5-7^. While cigarette smokers may use ECs to help them quit smoking^8-10^, and long-term EC users report that ECs helped them to cut down on smoking or quit^11^, the potential for youth to become addicted to nicotine in ECs is concerning^12^. In Malaysia, 0.8% of people aged 15 and older used ECs in the previous month in 2011^13^. In 2016, 3.2% of Malaysians aged ≥18 years used ECs in the previous month^14^. By 2019, prevalence among Malaysians aged ≥15 years reached 4.9%^15^. In that same year, 7.5% of Malaysian youth aged 15–19 years and 14.7% of young adults aged 20–24 years used ECs in the previous month compared with less than 5% of adults aged ≥30 years^15^.

EC use is more common among current smokers. In 2014, 11.5% of Malaysian smokers who had heard of ECs used them at least monthly, similar to the percentage of smokers from Canada (12.0%) and the US (11.8%) using ECs at least monthly in 2016, but lower than the percentage of smokers from England (17.2%) using ECs at least monthly^16^. Moreover, 75% of Malaysian EC users also smoked cigarettes in 2016^14^. Even though the sale of nicotine has been regulated in Malaysia since 1952 under the Poisons Act^17^, EC sales in Malaysia increased from US$106 million in 2012 to US$514 million in 2015^18^. Sales fell to US$229 million by 2016 following a 2015 national ban on nicotine containing e-liquids and subsequent bans on vaping in several Malaysian states^18^. To provide a recent snapshot of EC use in Malaysia, this study estimated the prevalence of ever, monthly, weekly, and daily EC use among Malaysian adults aged ≥18 years using data from the 2020 (Wave 1) International Tobacco Control (ITC) Malaysia (MYS1) Survey. It also assessed the types of EC products and flavors used by adult Malaysian cigarette smokers who reported using ECs in 2020.

## METHODS

The ITC MYS1 Survey was a cross-sectional, online survey conducted from 5 February to 3 March 2020. Respondents were quota sampled from an existing Rakuten Insight web panel that was nationally representative of Malaysian internet users. Rakuten Insight enrolls panelists daily from online sources to maintain a panel as consistent as possible with the general population^19^. Rakuten Insight collects detailed demographic information from its panelists that was used to invite them to the ITC MYS1 Survey. Invited panelists were screened to ensure they met inclusion criteria (aged ≥18 years; current smokers who smoked at least 100 cigarettes in their lifetimes and currently smoked at least monthly; non-smokers who had not smoked 100 cigarettes in their lifetimes or who had quit more than five years ago).

ITC MYS1 Survey questionnaire items were developed in English and translated into Malay and Chinese. Translations were checked to ensure accuracy and equivalence with the English content. Questionnaire items were developed from the larger ITC Project that has conducted cohort surveys of tobacco use since 2002^20,21^. Data quality control checks were conducted on the initial set of respondents (n=1313)^19,22^. Those who completed the survey too quickly (i.e. had an average response time of ≤1.93 seconds per item, which does not allow for reading the question) or who had an unusually high proportion of ‘don't know’ or ‘refused’ responses were excluded (n=60; final sample size=1253). Complete details of the ITC MYS1 Survey are described elsewhere^19,22^.

Daily smokers reported smoking every day while non-daily smokers reported smoking at least monthly. Non-smokers reported never smoking at all while former smokers reported quitting more than five years ago. No respondents reported quitting more recently than five years ago. Sampling weights were computed using a raking algorithm and calibrated to estimated population sizes^19,22^.

All respondents were asked whether they ever used an EC, even once. Respondents answering ‘Yes’ were classified as ever users. Respondents who ever used were asked whether they currently used ECs ‘Daily’, ‘Less than daily but at least once a week’, ‘Less than weekly, but at least once a month’, ‘Less than monthly’, and ‘Not at all’. Mutually exclusive categories of EC use were used to estimate prevalence of EC use (daily use, weekly use, or monthly use) and to assess the types and flavors of EC products used by cigarette smokers who used ECs at least monthly.

Sociodemographic measures were sex (male, female), age group (18–24, 25–39, 40–54 and ≥55 years), marital status (single, married/cohabiting, separated/widowed/divorced), ethnicity (Malay, Chinese, other), education level (secondary school or less, diploma/certificate, and Bachelor’s degree or higher), and employment status (employed full/part-time, otherwise). Sociodemographic measures were classified according to previous studies of tobacco use in Southeast Asia^23-25^, although education level was classified so that approximately one-third of respondents were assigned to each of the three categories^26^. Among smokers using ECs, characteristics examined were smoking status, cigarettes smoked/day, EC device type, whether their EC contained nicotine, perceived level of addiction to ECs, and EC flavors used in the previous month. Data were analyzed using the survey procedures in SAS software (Version 9.4, SAS Institute Inc., Cary, NC, USA) to account for the stratified design and sampling weights. Prevalence was estimated using inflation weights while characteristics of smokers using ECs were estimated using rescaled weights^22^; exact 95% confidence intervals were estimated for all percentages^27^. Rao-Scott χ^2^ tests and logistic regression tested differences in the characteristics of EC users by frequency of use and a Bonferroni correction adjusted for multiple comparisons.

## RESULTS

Overall, 33.7% of Malaysians reported ever using ECs (95% CI: 28.0–39.8), 2.3% used ECs monthly (95% CI: 1.4–3.5), 3.7% used ECs weekly (95% CI: 2.8–5.0), and 5.4% used ECs daily (95% CI: 3.7–7.5) (Table 1). Daily EC use was more common among current cigarette smokers (17.4%) than non-smokers (0.6%), among males (10.3%) than females (0.3%), and among Malaysians who were currently employed (6.8%) than among those who were not employed (2.0%).

**Table 1:** Prevalence of e-cigarette use among Malaysian adults aged ≥18 years in 2020 (N=1253, weighted estimates ^a^)

*Characteristics*	*n*	*Ever used*		*Monthly*		*Weekly*		*Daily*	
		*%*	*95% CI*	*%*	*95% CI*	*%*	*95% CI*	*%*	*95% CI*
**Overall**	1253	33.7	28.0–39.8	2.3	1.4–3.5	3.7	2.8–5.0	5.4	3.7–7.5
**Cigarette smoking status**									
Daily	887	75.2	71.3–78.8	7.1	5.1–9.4	15.8	12.7–19.2	17.4	14.6–20.4
Non-daily	160	72.2	62.2–80.8	6.0^c^	2.2–12.6	14.8	9.3–21.9	20.0	12.5–29.5
Former smoker	49	24.7	12.6–40.6	–^b^		–^b^		5.1^c^	0.6–17.1
Non-smoker	157	20.7	13.0–30.2	1.2^c^	0.1–4.5	0.4^c^	0.0–3.1	0.6^c^	0.0–3.4
**Gender**									
Male	1024	50.3	42.1–58.4	3.3	2.3–4.6	6.8	5.2–8.7	10.3	7.0–14.4
Female	229	16.6	9.2–26.6	1.2^c^	0.1–4.2	0.6^c^	0.0–2.7	0.3	0.0–2.2
**Age** (years)									
18–24	180	34.5	19.2–52.5	3.7^c^	0.7–10.7	3.4	1.3–7.2	5.0	2.3–9.3
25–39	749	34.0	26.2–42.4	1.6	0.8–2.8	3.8	2.5–5.4	5.3	3.0–8.6
40–54	265	39.8	27.7–52.8	3.4^c^	1.3–7.0	4.9	2.6–8.2	6.5^c^	2.5–13.2
≥55	59	8.5^c^	2.8–18.7	– ^b^		0.5^c^	0.0–7.0	2.7^c^	0.2–10.7
**Marital status**									
Single	445	35.6	25.8–46.4	2.8^c^	1.1–5.8	3.4	1.9–5.6	4.4	2.7–6.8
Married/cohabiting	744	31.9	24.8–39.8	1.8	1.0–3.1	3.8	2.5–5.4	6.0	3.5–9.5
Separated/divorced/ widowed	59	51.6	25.8–76.8	5.6^c^	1.3–14.9	8.4^c^	2.3–20.2	4.0^c^	0.6–12.7
**Ethnicity**									
Malay	619	35.5	27.2–44.6	1.5	0.7–2.8	3.2	1.9–4.9	6.1	3.7–9.5
Chinese	461	21.2	15.2–28.4	2.5^c^	0.5–7.1	2.8^c^	1.3–5.2	3.4^c^	1.4–6.8
Other	173	50.0	31.9–68.2	5.6^c^	2.1–11.7	8.6	4.4–14.7	5.8^c^	2.8–10.4
**Education level**									
Secondary or less	404	37.5	27.6–48.2	3.6^c^	1.5–7.3	5.9	3.7–8.9	4.3	2.5–6.8
Diploma/certificate	362	30.4	21.5–40.4	1.6	0.6–3.5	2.5	1.1–4.6	6.8	3.5–11.8
Bachelor’s degree or higher	480	30.7	21.2–41.7	1.6^c^	0.7–3.2	3.1	1.7–5.1	4.7^c^	1.9–9.5
**Employment status**									
Employed full/part-time	1055	36.1	29.6–43.1	3.0	1.8–4.7	4.7	3.5–6.2	6.8	4.5–9.8
Otherwise	198	27.8	16.6–41.5	0.4^c^	0.0–2.7	1.4^c^	0.3–4.2	2.0	0.5–5.0

a Weighted estimates from the 2020 (Wave 1) ITC Malaysia Survey.

b No respondents in this group reported using EC.

c High sampling variability (relative standard error ≥0.3); interpret with caution.

Among current cigarette smokers who used ECs at least monthly, 88.3% (95% CI: 84.6–91.4) were daily smokers who smoked 13.0 cigarettes per day (95% CI: 12.1–14.0), irrespective of frequency of EC use. Slightly more than half of weekly (54.3%) and monthly (51.4%) users reported using pre-filled ECs while daily users equally preferred pre-filled (46.2%) and tank (43.7%) devices (Table 2). A significantly greater percentage of daily (81.0%) than monthly (62.5%; p=0.045) users reported their ECs contained nicotine. A significantly greater percentage of daily users (60.4%) reported being somewhat/ very addicted to ECs compared with weekly (36.1%; p<0.001) or monthly (21.9%; p<0.001) users. The most common EC flavors were fruit, coffee, and menthol/mint, irrespective of frequency of use. Clove-flavored ECs were used by less than 20% of all users.

**Table 2:** Characteristics of and e-cigarette products used by Malaysian adult cigarette smokers who reported using e-cigarettes at least monthly in 2020 by frequency of e-cigarette use (N=459, weighted estimates ^a^)

*Characteristics*	*Frequency of e-cigarette use*						*p ^c^*
	*Daily (n=212)*		*Weekly (n=177)*		*Monthly (n=70)*		
	*%*	*95% CI*	*%*	*95% CI*	*%*	*95% CI*	
**Smoking status**							
Daily	86.9	80.3–91.9	89.1	83.6–93.3	90.1	79.7–96.3	0.749
Non-daily	13.1	8.1–19.7	10.9	6.7–16.4	9.9^b^	3.7–20.3	
**Cigarettes/day, daily smokers only**							
Mean	13.4	12.1–14.8	12.5	11.0–14.0	13.2	11.1–15.3	0.669
**EC device type**							
Disposable	10.1	6.3–15.1	12.8	6.9–21.1	9.5^b^	3.0–21.2	0.732
Pre-filled	46.2	37.8–54.7	54.3	44.0–64.4	51.4	36.8–65.9	0.458
Tank system	43.7	35.3–52.4	32.5	23.6–42.5	37.2	24.0–51.9	0.205
Other	-	-	0.4^b^	0.0–2.8	2.0^b^	0.1–8.7	^d^
Nicotine in EC^e^	81.0^x^	72.5–87.7	73.3^xy^	64.2–81.2	62.5^y^	47.6–75.8	0.044
Somewhat/very addicted to EC^e^	60.4^x^	51.9–68.6	36.1^y^	27.0–46.0	21.9^y^	11.6–35.6	<0.001
**Flavor**							
Fruit	88.8	82.9–93.2	82.2	72.3–89.8	76.2	62.4–86.9	0.102
Coffee	60.0	51.3–68.3	58.4	48.3–68.1	59.5	45.4–72.5	0.968
Menthol/mint	54.9	46.4–63.3	55.7	45.0–66.0	58.3	43.6–72.0	0.925
Candy	52.3	43.7–60.8	45.4	35.4–55.8	53.9	39.4–68.0	0.484
Tobacco	48.3	39.9–56.8	47.8	37.6–58.1	43.0	29.3–57.7	0.814
Non-alcoholic beverage	32.1	24.5–40.4	28.2	19.0–39.0	30.9	18.2–46.2	0.828
Chocolate	28.9	22.0–36.6	36.7	27.1–47.3	33.7	20.7–48.8	0.442
Clove	15.7	10.6–21.9	20.6	13.6–29.3	13.2^b^	5.2–26.1	0.415
Unflavored	12.2	7.7–18.1	23.3	14.9–33.7	19.3^b^	9.0–33.9	0.090
Another flavor	9.5	5.2–15.7	10.9^b^	4.2–21.8	11.1^b^	3.7–24.3	0.941

a Weighted estimates from the 2020 (Wave 1) ITC Malaysia Survey.

b High sampling variability (relative standard error ≥0.3); interpret with caution.

c p-value from a Rao-Scott χ² test for the association between frequency of e-cigarette use and each outcome. For mean CPD, p-value from an F-test from a linear regression model to test the difference in average CPD between EC user groups.

d No daily users reported using ‘other’ types of EC; as a result, the association between frequency of use and type of EC use could not be tested.

e Pairwise differences between e-cigarette frequency of use groups were tested using logistic regression; different superscript letters (x, y) indicate statistically significant differences between groups after correcting for multiple testing using a Bonferroni adjustment.

## DISCUSSION

In 2020, 11.4% of Malaysian adults aged ≥18 years used ECs at least once a month and 5.4% used ECs daily, representing increases of 8.2 and 4.6 percentage points, respectively, since the 2016 National E-Cigarette Survey (NECS)^14^. Estimates from 2020 are also higher than those from the 2019 Malaysian National Health and Morbidity Survey (NHMS). However, the prevalence of at least monthly EC use among young adults aged 20–24 years was comparable to the NHMS (ITC MYS1=11.4%, 95% CI: 5.9–19.5; NHMS=14.7%, 95% CI: 9.9–21.3)^15^. Furthermore, among daily cigarette smokers from Malaysia and England, the prevalence of daily EC use in Malaysia in 2020 was higher than the prevalence of at least monthly EC use in England in 2016 (17.4% vs 13.7%, respectively)^16^. This finding is striking because England has less restrictive EC policies than many other countries^16^, and because 40% of cigarette smokers in England try to quit smoking using ECs^28^. In summary, the prevalence of EC use in Malaysia appears to be higher than in other countries with less restrictive EC policies.

Most cigarette smokers who used ECs daily reported their device contained nicotine and being addicted to ECs. Dual use of cigarettes and ECs may contribute to continued nicotine addiction. However, most Malaysian smokers who use ECs daily report using them to quit (88%) or cut down on the number of cigarettes they smoke (91%)^29^. It is reassuring that EC use is rare among Malaysian non-smokers, suggesting that most adult Malaysian non-smokers have not started using these products.

### Limitations

While these findings present a snapshot of EC use in Malaysia in 2020, limitations of the ITC MYS 1 Survey must be considered. First, it is not possible to determine whether EC use increased in Malaysia. Unlike the 2019 NHMS, the 2020 ITC MYS 1 Survey sampled from a non-probability commercial web panel. Although that panel was representative of internet penetration, it is possible that some groups were over-represented (younger, urban Malaysians) while others were under-represented (older, rural Malaysians). This might account for differences in prevalence between surveys. Second, some estimates presented here are based on small sub-samples resulting in high sampling variability. These estimates must be interpreted cautiously.

## CONCLUSIONS

Current EC use is most common among cigarette smokers in Malaysia. Longitudinal studies of Malaysian smokers are needed to determine whether EC use influences cigarette smoking cessation. Continued surveillance of EC use in Malaysia is needed to monitor whether EC initiation rates change among youth, young adults, and non-smokers.

## Data Availability

The data supporting this research are available from the authors on reasonable request.
